# A novel retinal ganglion cell quantification tool based on deep learning

**DOI:** 10.1038/s41598-020-80308-y

**Published:** 2021-01-12

**Authors:** Luca Masin, Marie Claes, Steven Bergmans, Lien Cools, Lien Andries, Benjamin M. Davis, Lieve Moons, Lies De Groef

**Affiliations:** 1 Department of Biology, Neural Circuit Development and Regeneration Research Group, KU Leuven, Leuven, Belgium; 2 Glaucoma and Retinal Neurodegenerative Disease Research Group, Institute of Ophthalmology, University College London, London, UK; 3 Central Laser Facility, Science and Technologies Facilities Council, UK Research and Innovation, Didcot, Oxfordshire UK

**Keywords:** Software, Cell death in the nervous system, Retina, Glaucoma, Neurodegeneration

## Abstract

Glaucoma is a disease associated with the loss of retinal ganglion cells (RGCs), and remains one of the primary causes of blindness worldwide. Major research efforts are presently directed towards the understanding of disease pathogenesis and the development of new therapies, with the help of rodent models as an important preclinical research tool. The ultimate goal is reaching neuroprotection of the RGCs, which requires a tool to reliably quantify RGC survival. Hence, we demonstrate a novel deep learning pipeline that enables fully automated RGC quantification in the entire murine retina. This software, called RGCode (Retinal Ganglion Cell quantification based On DEep learning), provides a user-friendly interface that requires the input of RBPMS-immunostained flatmounts and returns the total RGC count, retinal area and density, together with output images showing the computed counts and isodensity maps. The counting model was trained on RBPMS-stained healthy and glaucomatous retinas, obtained from mice subjected to microbead-induced ocular hypertension and optic nerve crush injury paradigms. RGCode demonstrates excellent performance in RGC quantification as compared to manual counts. Furthermore, we convincingly show that RGCode has potential for wider application, by retraining the model with a minimal set of training data to count FluoroGold-traced RGCs.

## Introduction

Glaucoma is a group of optic neuropathies and one of the most researched ophthalmological pathologies. Major hallmarks of this disease include optic nerve damage and retinal ganglion cell (RGC) death, yet a grand unifying theory explaining the underlying pathophysiology has not yet been realised. As a consequence, current glaucoma therapies target the only known treatable risk factor—elevated intraocular pressure—, rather than actively impeding the underlying pathological mechanisms. The unmet clinical need for new glaucoma medication shifted the focus of researchers towards neuroprotection—i.e. preventing the loss of RGCs.

In preclinical glaucomatous research, RGC survival is the primary efficacy measure of neuroprotective therapies in rodent models. Survival—or inversely, loss—of RGC somata has been evaluated by a number of methods; post mortem via retrograde tracing and immunohistochemical stainings^1^ or in vivo with the real-time imaging technology DARC (Detection of Apoptosing Retinal Cells)^2^. Before the identification of RGC specific antibodies, retrograde labelling was considered the gold standard technique for RGC quantification. Commonly used tracers include FluoroGold/hydroxystilbamidine, DiI, Cholera toxin B or dextran tetramethylrhodamine, which are applied directly to the optic nerve or to regions of the brain to which RGCs project, such as the superior colliculus^3^. Limitations of this technique, however, include requiring invasive surgical procedures to introduce the tracer and the labelling of a subset of RGCs after tracer administration at brain target areas, as only RGCs projecting to that area will be labelled^1^. Moreover, the localisation of the tracer is not confined to RGCs as the dye is reported to translocate to microglia^4,5^, although efforts can be made to exclude these based on morphology. Last, next to RGC loss, absence of the tracer can also denote impaired axonal transport or axon degeneration rather than absence of RGCs, which further complicates data interpretation^1^.

Immunohistochemical labelling of RGC specific molecular markers is currently the favoured method for assessing RGC survival. Brain-specific homeobox/POU domain protein 3A (Brn3a) was a widely utilised pan-RGC marker. Chosen for its high specificity and being a nuclear restricted label—meaning Brn3a+ RGCs are extremely well resolved, even in regions of high RGC density—Brn3a labelling enabled the development of the first generation of automated RGC population counting algorithms. More recently, however, several limitations of Brn3a as an RGC marker have been identified. Firstly, Brn3a expression is reported to acutely decrease when RGCs are under stress but still alive, resulting in an overestimation of the actual RGC death in animal glaucoma injury models^6–8^. Moreover, Brn3a is shown to label only 80% of the total RGC population, excluding disease relevant subtypes such as the melanopsin expressing RGCs^7,8^. With the introduction of the novel marker RNA-binding protein with multiple splicing (RBPMS), Brn3a was replaced by RBPMS as the go-to marker to count RGCs. Indeed, recent single cell transcriptomics experiments confirm a uniform expression of the RBPMS gene in all RGC subtypes, whereas the expression of Brn3a is highly inconsistent and barely present in a minor number of subtypes^9,10^. Unfortunately, existing Brn3a counting routines cannot be easily adapted to RBPMS labelling due to the nature of labelling: nuclear (Brn3a) versus cytoplasmic (RBPMS) staining^11^. Automated counting of cytoplasmic RGC labelling is complicated due to the fact that the many RGCs subtypes—at least 46 in rodents^9,10,12,13^—cover a wide range of somata sizes, from 10 to 35 $\upmu$m in diameter^8^. This is in stark contrast to a nuclear RGC staining, which has the advantage of resulting in a more consistent labelling in terms of size as well as circularity and brightness, rendering it more amenable for automatic quantification. Indeed, the majority of research articles studying RBPMS-immunopositive (RBPMS+) RGCs are reporting manual counts^11,14–16^, still resulting in a lack of a fully automated pipeline that does not require entering user-defined variables, although attempts are being made with classic image analysis techniques^8,17^. Fully automated RBPMS labelling requires to go beyond conventional automatic counting methods and look towards artificial intelligence.

Deep learning-based approaches for image analysis, especially convolutional neural networks (CNNs), have recently gained a lot of attention in biology as well as other fields^18^. In particular, U-Net-based CNN architectures are being increasingly used for medical and biological semantic image segmentation^19^, also in the ophthalmology research field^20^. U-Net is a fully convolutional encoder-decoder network, meaning that it can output semantic feature representations from raw image inputs and allows for precise localisation of the features^19^. While initially conceived for image segmentation, the U-Net architecture can be adapted to counting tasks^21,22^. Here, we present such an example: RGCode, a U-Net-based approach for the automated detection and quantification of murine RBPMS+ RGCs. RGCode was trained using naive and glaucomatous retinas to account for possible injury associated changes in RGC morphology and labelling. Two glaucoma injury paradigms were used: the mild microbead-induced ocular hypertension (OHT) and the more severe optic nerve crush (ONC). The counting program requires the input of entire RBPMS-immunostained retinal flatmounts imaged with a wide-field microscope, thus eliminating the need for more time-consuming and costly confocal images. The program is fully automated and trained to be robust to inter- and intra-experiment variation in immunohistochemical staining. As such, it does not necessitate preprocessing of the images to remove artefacts, outlining the retina nor adjusting the brightness/contrast. Moreover, testing RGCode on different datasets (random brightness differences, confocal images and FluoroGold-traced RGCs), shows that the program has a broad applicability without the need—or with a minimal set—of additional training data. The algorithm described in this manuscript is provided with a user-friendly interface and in an accessible format, available at https://gitlab.com/NCDRlab/rgcode.

## Results

### Dataset design

**Figure 1:**
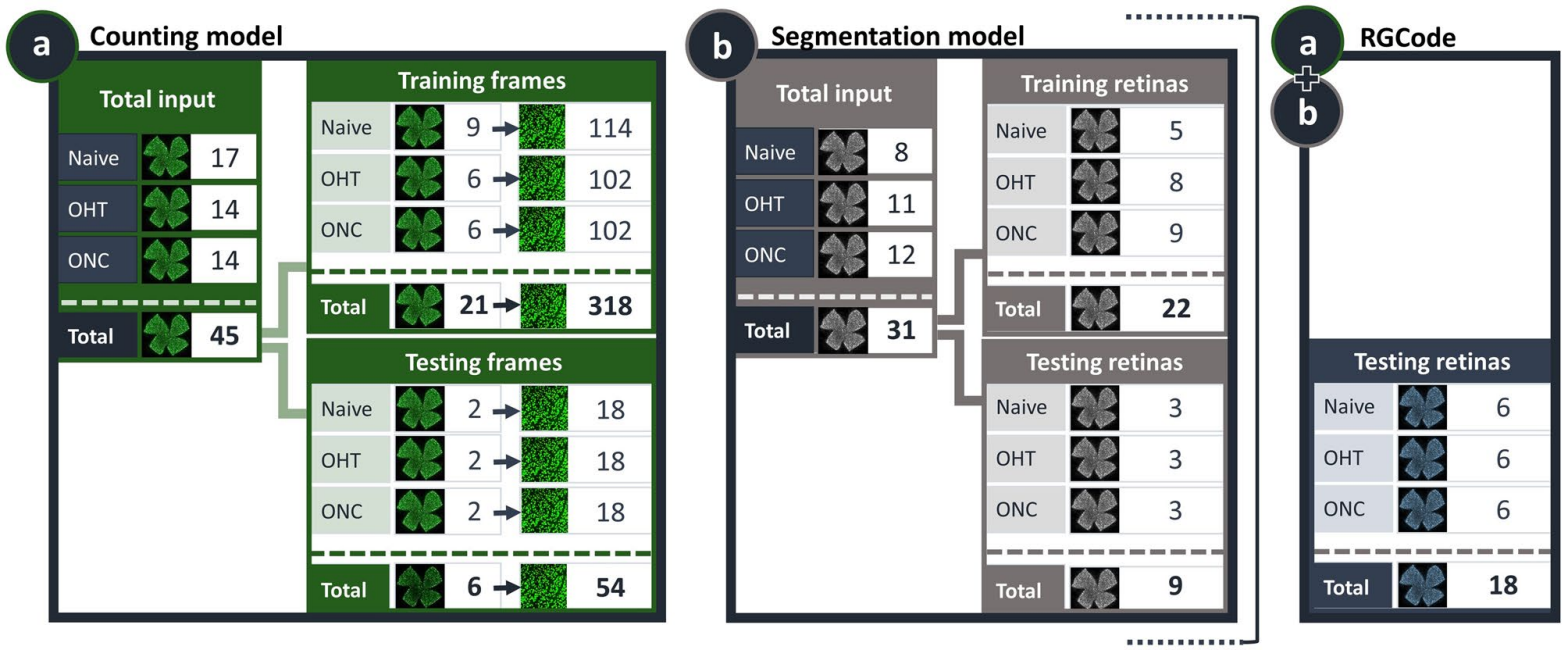
Overview of the datasets used to develop RGCode. Composition of the training and testing datasets for both the counting (**a**) and segmentation (**b**) models are depicted. Frames were sampled from different retinal areas and experimental conditions (naive, OHT- and ONC-injured retinas). Additionally, 18 entire flatmounts were reserved for evaluating the performance of the complete pipeline, comprising both the counting and segmentation algorithms (**a+b**). Key: Naive, uninjured retinas; OHT, 5 weeks post microbead-induced ocular hypertension retinas; ONC, 7 days post optic nerve crush injury retinas.

The presented deep-learning based method for RGC quantification is based on two U-Net models to fully automate RGC counting (counting model) and segmentation of retinal area (segmentation model) on RBPMS-stained flatmounts. The combination of both models results in “RGCode”, an algorithm to quantify and visualise (isodensity maps) RGC density. In order to train the models as robust as possible, we created a curated dataset comprising RBPMS-stained retinas across three different conditions: (i) uninjured, naive retinas, (ii) retinas from the microbead-induced OHT paradigm, to represent a mild glaucoma-like RGC pathology, and (iii) retinas harvested after ONC, to represent severe RGC loss. All samples were collected, processed and imaged in two batches to account for experimental variation. Retinas were divided into two datasets: a training one—from which the model can learn to recognise RGCs (counting model) and retinal boundaries (segmentation model)—and a testing one—to unbiasedly assess the performance of the resulting models on new, unseen images. A schematic overview of the image subsets, along with their sample sizes is given in Fig. 1.

The total amount of training data fed to the counting model was 318 frames (354 $\times$ 354 $\upmu \hbox {m}$), which were sampled from every region of 21 RBPMS-stained uninjured and glaucomatous retinas (Fig. 1a), including boundary cases (Supplementary Fig. S1). This corresponded to a total of 91,993 RBPMS+ cells and a coverage of approximately 10% of the retinal area per retina. The training frames were divided amongst three human experts, who manually annotated RBPMS+ cells according to preestablished counting rules (Supplementary Fig. S2). To train the segmentation model, 22 retinas were manually outlined and fed as input (Fig. 1b). Of note, image augmentation techniques were used during training to further increase robustness whilst keeping a manageable dataset size (cfr. Methods). Conversely, the testing dataset of the counting model comprised 54 frames sampled from 6 retinas (a total of 15128 annotated cells), across the three different conditions (Fig. 1a). Cells were counted in triplicate by all human experts to account for inter-operator variability and to compare our model to human bias. The segmentation performance was evaluated against 9 manually outlined retinas, 3 for each experimental condition (Fig. 1b). Finally, 6 retinas per experimental condition were reserved to compare the outcome of RGCode to the literature (Fig. 1a+b). The statistical analysis of the performance in each task will be discussed in detail in the following sections.

### Performance of the counting model

**Figure 2:**
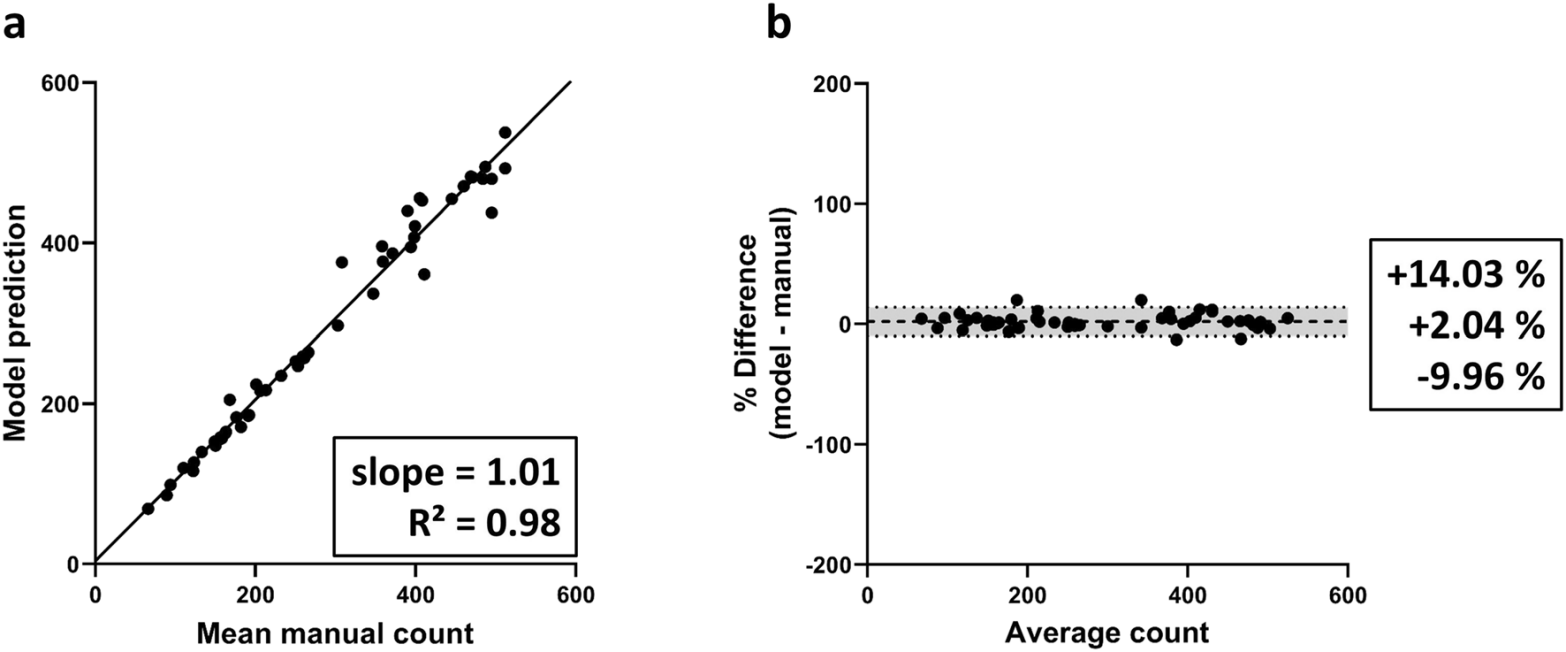
Linear regression and bias analysis of automated counts on retinal frames. (**a**) Linear regression analysis for the average of the manual counts versus model output indicates that the automated counts show a very high linear relationship with manual counts. Best-fit linear regression correlation coefficient ($\hbox {R}{^2}$ and slope) are indicated. (**b**) Bland–Altman analysis of the automated counts compared to the operator average, showing mean bias $\pm 95\%$ limits of agreement.

**Figure 3:**
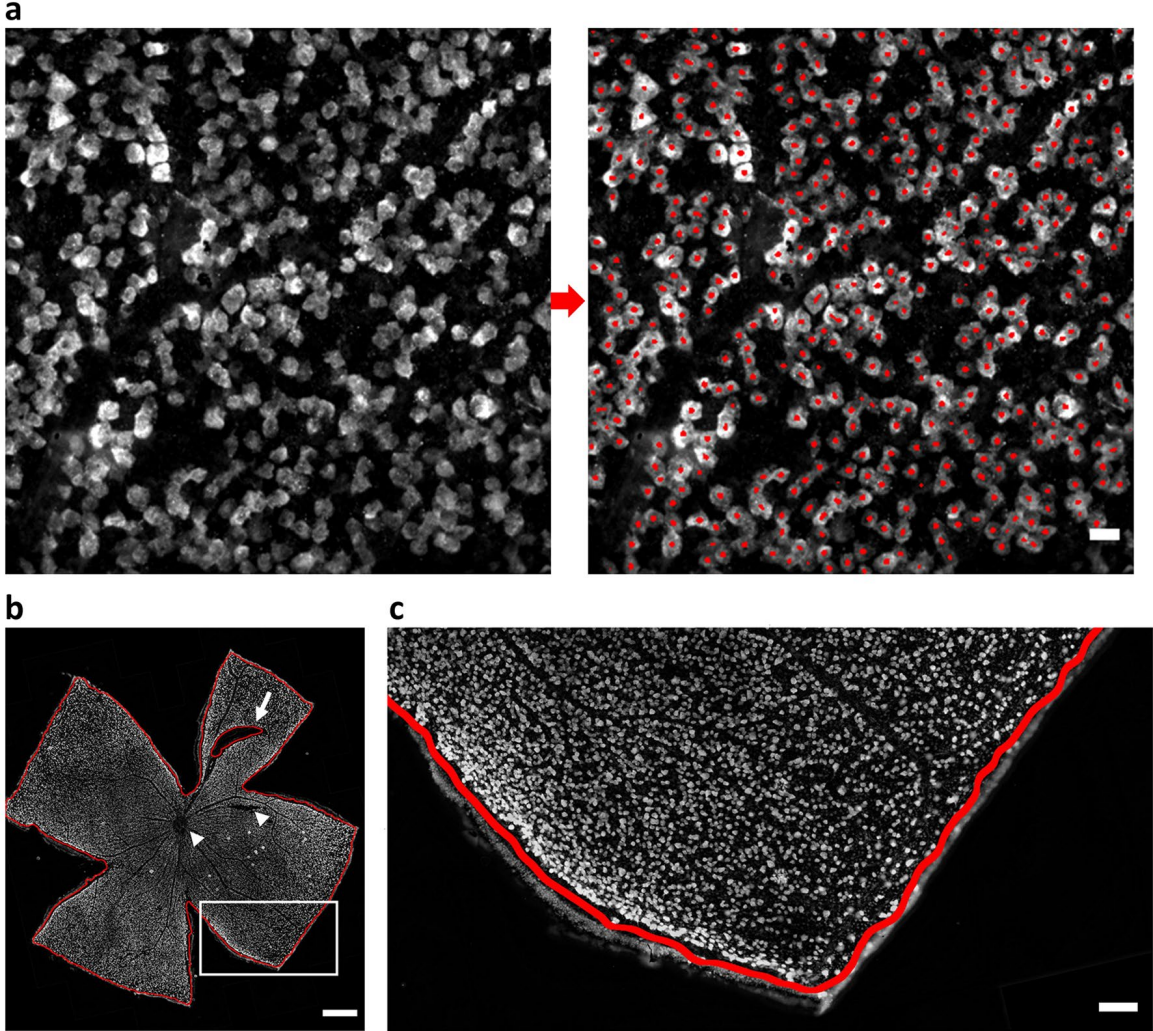
Representative examples of the output of RGCode on testing data. (**a**) Testing frame sampled from the mid-peripheral region of a naive retina, showing the input and output of RGCode. In general, RGCode consistently detects and counts RBPMS+ cells and clearly distinguishes overlapping cells. Scale bar $= 20 \,\upmu \hbox {m}$. (**b**, **c**) Naive testing retina showing the output of the segmentation model. Scale bars = 500 $\upmu \hbox {m}$ and $100 \,\upmu \hbox {m}$ (zoom).

To study the performance of our counting algorithm, the average of operator counts was compared to the resulting predictions by RGCode. Firstly, we evaluated the correlation between both counting methods, using the Intraclass Correlation Coefficient (ICC) as a metric. The trained model showed an excellent correlation with the average of the operator counts, revealing an ICC of 0.988, with 95% limits of agreement ($\hbox {CI}_{95\%}$) [− 0.008, + 0.004]. This correlation is comparable to the inter-observer correlation, which showed an ICC of 0.979, with $\hbox {CI}_{95\%}$ [− 0.013, $+$ 0.008]. Secondly, linear regression analysis showed an overall slope of best linear fit of 1.01 and an $\hbox {R}{^2}$ of 0.98, denoting a strong correlation with the mean manual counts and good performance across all retinal regions (Fig. 2a). Comparable performance was recorded on each condition separately (naive, OHT- and ONC-injured retinas), with a slope and $\hbox {R}{^2}$ close to 1 for all (Supplementary Fig. S3). To further asses the agreement between automated and manual counts, Bland–Altman plots were generated. When compared to the average manual counts, the model showed an insubstantial bias of + 2.04% with $\hbox {CI}_{95\%}$ of [− 9.96, + 14.03%] compared to operator counts (Fig. 2b). Notably, when comparing human counters with each other, comparable biases were measured (Supplementary Fig. S4). Biases for the automated counts on naive, OHT- and ONC-injured frames singularly were comparable to each other (respectively + 2.94, + 0.65 and + 2.53% with $\hbox {CI}_{95\%}$ [− 12.46, + 18.34%], [− 6.08, + 7.37%] and [− 9.88, + 14.94%], Supplementary Fig. S3). Taken together, these results indicate that the model performs equally well across all injury paradigms and its reliability is comparable to an experienced human operator. Representative outputs of automated RGC quantification on testing frames of each experimental condition are shown in Fig. 3a and Supplementary Fig. S5.

### Performance of the segmentation model

**Figure 4:**
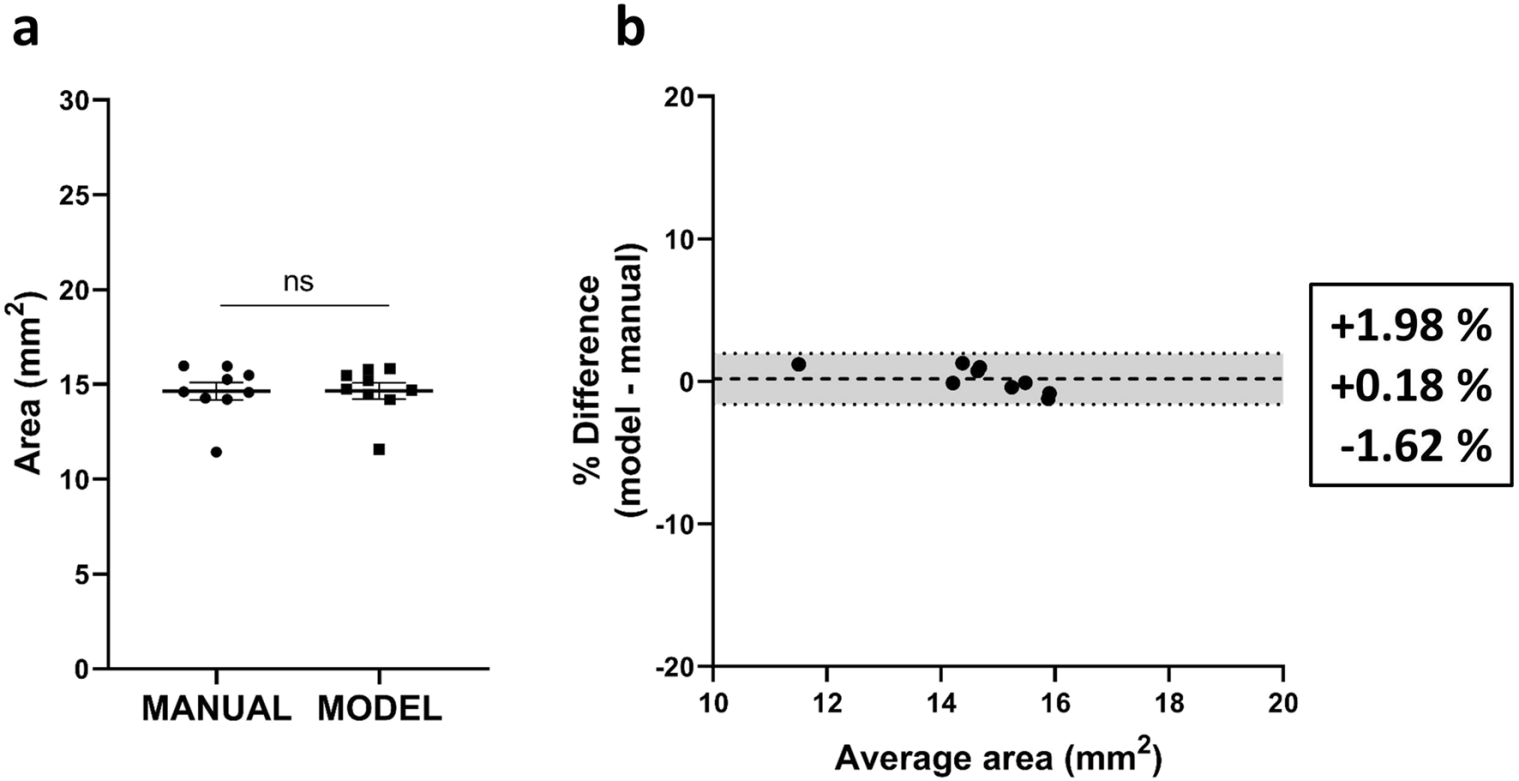
Estimation of retinal area and bias analysis of automated segmentation on retinal flatmounts. (**a**) Comparison of manual and automated segmentation of the retinal area (mean ± SEM) revealing no significant difference between both methods (unpaired two-tailed *t* test). (**b**) Bland–Altman analysis of the automated versus manual segmentation. Mean bias ± 95% limits of agreement are shown.

In order to have a fully automated pipeline to assess RGC densities on entire flatmounts, we developed a segmentation model to automatically measure the retinal area, eliminating the requirement of manual outlining. To determine the segmentation performance, we analysed the Jaccard index or “Intersection over Union” (IoU). The IoU is a measurement of agreement between segmentations calculated by dividing the area of overlap of the masks by their sum. As such, an outcome of 1 indicates a complete agreement between the manual and the computed segmentation. Our segmentation model performed very well, obtaining an average IoU of $0.968 \pm 0.001$, indicating a high rate of overlap between the segmentations. Indeed, the mean of the measured areas was not statistically different between manual and automated counts, showing a mean retinal area of $14.64 \pm 0.46$ and $14.66~\pm ~0.43 \hbox {mm}{^2}$ for manual and automated segmentation respectively (Fig. 4a). Bland–Altman analysis of automated versus manual segmentation showed an insubstantial bias of + 0.18%—equivalent to $0.019\hbox { mm}{^2}$—, with $\hbox {CI}_{95\%}$ [$\,-$1.62, + 1.98%] (Fig. 4b). Representative outputs of automated retinal segmentation on a testing frame is shown in Fig. 3b,c.

### Performance of RGCode

**Figure 5:**
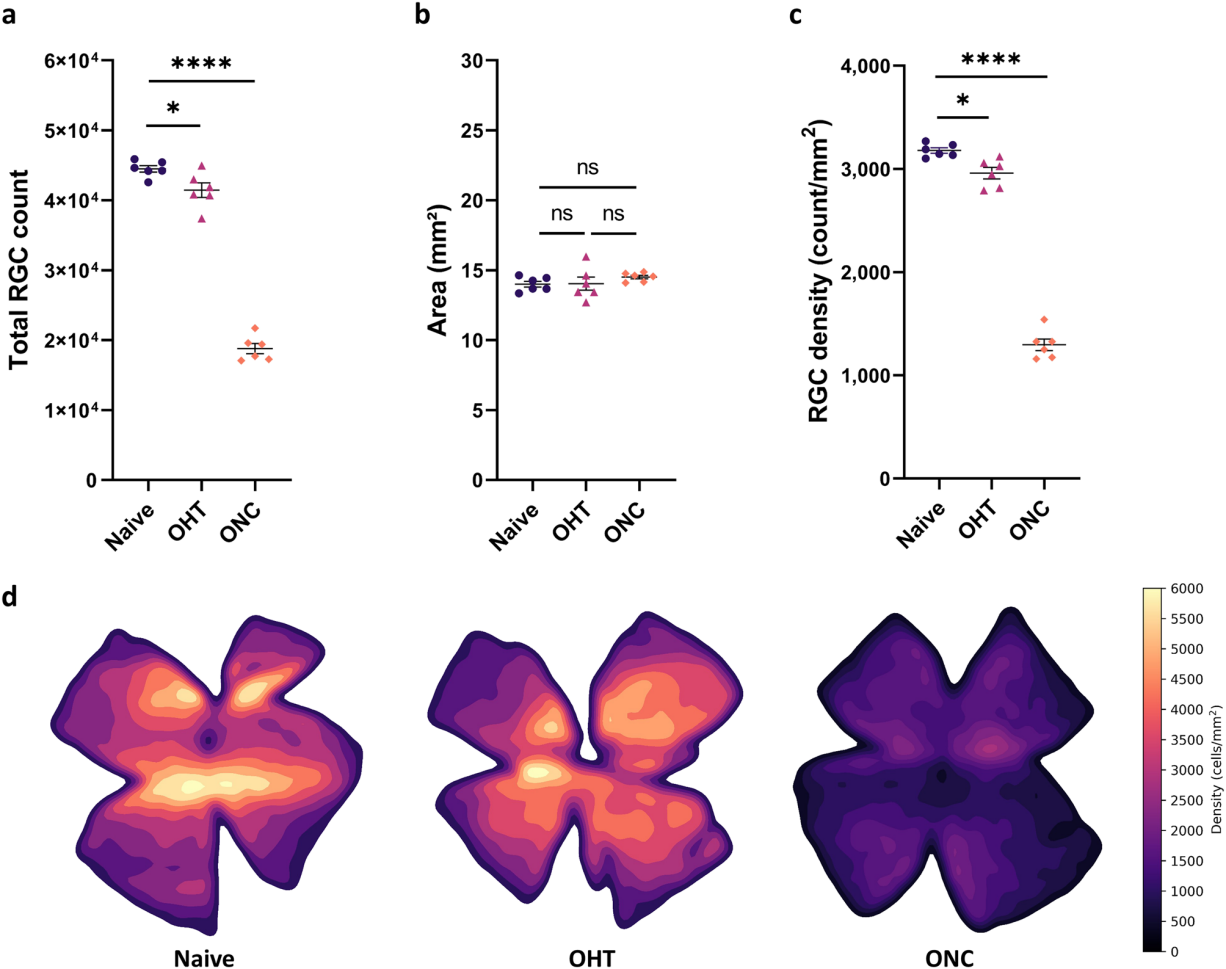
Outcome of RGCode on testing retinas: automated RGC count, retinal segmentation, RGC density and isodensity maps across all conditions (Naive, purple dots; OHT, pink triangles and ONC, orange squares). (**a**) A significant decrease in RGC count is seen in glaucomatous conditions as opposed to uninjured retinas (one-way ANOVA with Tukey’s post-hoc test, $$*{p }= 0.0372$$, $$****{p }< 0.0001$$). (**b**) The total area does not significantly differ between uninjured and injured retinas (one-way ANOVA with Tukey’s post-hoc test). (**c**) RGC density is significantly lower when comparing glaucomatous versus uninjured retinas (one-way ANOVA with Tukey’s post-hoc test, $$*{p} = 0.0145$$, $$****{p} < 0.0001$$). Data are depicted as mean ± SEM. (**d**) Representative pseudo-colour representations ranging from 0 RGCs/mm$${^2}$$ (black tone) to 6000 RGCs/mm$${^2}$$ (yellow tone) of uninjured and injured (OHT and ONC) retinas. A central-to-peripheral gradient in RGC density can be observed in the uninjured retina. Modest (OHT) and substantial (ONC) RGC loss is detected in the glaucomatous retinas. Key: Naive, uninjured retinas; OHT, 5 weeks post microbead-induced ocular hypertension retinas; ONC, 7 days post optic nerve crush injury retinas.

The two U-Net models were integrated in a fully automated software program for RGC counting and density measurements of entire flatmount retinas (RGCode; https://gitlab.com/NCDRlab/rgcode). The software is accessible both via a user-friendly interface and via command-line, for integration in downstream automated pipelines. After inputting retinal flatmounts with RBPMS labelled RGCs, RGCode will operate in batch mode to count the RGCs and segment the retina. RGCode returns both the predicted binary masks for control and/or further processing, and an Excel file indicating RGC count, RGC density and area per retina. Optionally, the program can generate retinal isodensity maps—i.e. pseudocolor representations of RGC density—, overlay pictures—in which the outline and the position of the detected RGCs is annotated—and cell coordinates—useful to further compute custom spatial statistics. Representative isodensity maps for each condition are shown in Fig. 5d. Of note, users can choose the resolution of the density estimation according to their preference, allowing a lesser or more detailed map. Moreover, the entire segmentation part of the pipeline can be skipped, if the user wishes to perform the automated counting on retinal frames only. A full analysis of one retina, with overlay and isodensity output, took around 10 min on a basic dual-core office laptop (Intel Core i5-7300U, 8 GB RAM). On an eight-core office workstation, one retina was analysed in around 4 minutes while using only CPU, or in 2:30 min if GPU acceleration was used (AMD Ryzen PRO 2700 CPU, 32GB RAM and Nvidia P1000 GPU). Running RGCode on the testing flatmounts revealed an average RBPMS+ RGC count of $44,499 \pm 470$ cells in uninjured retinas, whereas OHT and ONC retinas have on average of $41,458 \pm 1038$ and $18,785 \pm 730$ RBPMS+ cells, respectively (Fig. 5a). The mean areas were on average $14.01 \pm 0.21$, $14.04 \pm 0.47$ and $14.51 \pm 0.13$ mm${^2}$ (Fig. 5b). Mean densities calculated by RGCode were $3,179 \pm 26$ (uninjured), $2,960 \pm 55$ (OHT) and $1,296 \pm 57$ (ONC) RBPMS+ cells/mm${^2}$, corresponding to a significant RGC loss of $6.89\% \pm 1.90$% at 5 weeks after microbead injection and $59.20\% \pm 1.96$% at 7 days post ONC injury (Fig. 5c).

### Testing and training RGCode on different datasets

To further test the robustness of RGCode, testing data were subjected to random brightness shifts from − 50 to + 50% in 6 different runs. Linear regression analysis of the average of the randomisation runs against the average of operator counts revealed a slope of best fit of 1.02 and an $\hbox {R}{^2}$ of 0.98 (Supplementary Fig. S6), practically indistinguishable from the performance on the non-randomised data. The same held true for the counting bias, which was 2.92% with $\hbox {CI}_{95\%}$ [− 9.15, $+$ 14.99%] (Supplementary Fig. S6). Next, RGCode was applied on retinas acquired with a different imaging technique; confocal microscopy (cfr. Methods). Of note, this resulted in images with a considerably different resolution (0.96 pixels/$\upmu \hbox {m}$) as compared to all other epifluorescence microscopy images used in this study (2.17 pixels/$\upmu \hbox {m}$). Nonetheless, RGCode performed very well; the recorded retinal area, RGC number and density for confocal retinal images were not statistically different as compared to images acquired on an epifluorescence microscope (Supplementary Fig. S7). As such, RGCode allows for different input files than what it was trained on, while maintaining its counting and segmentation robustness.

**Figure 6:**
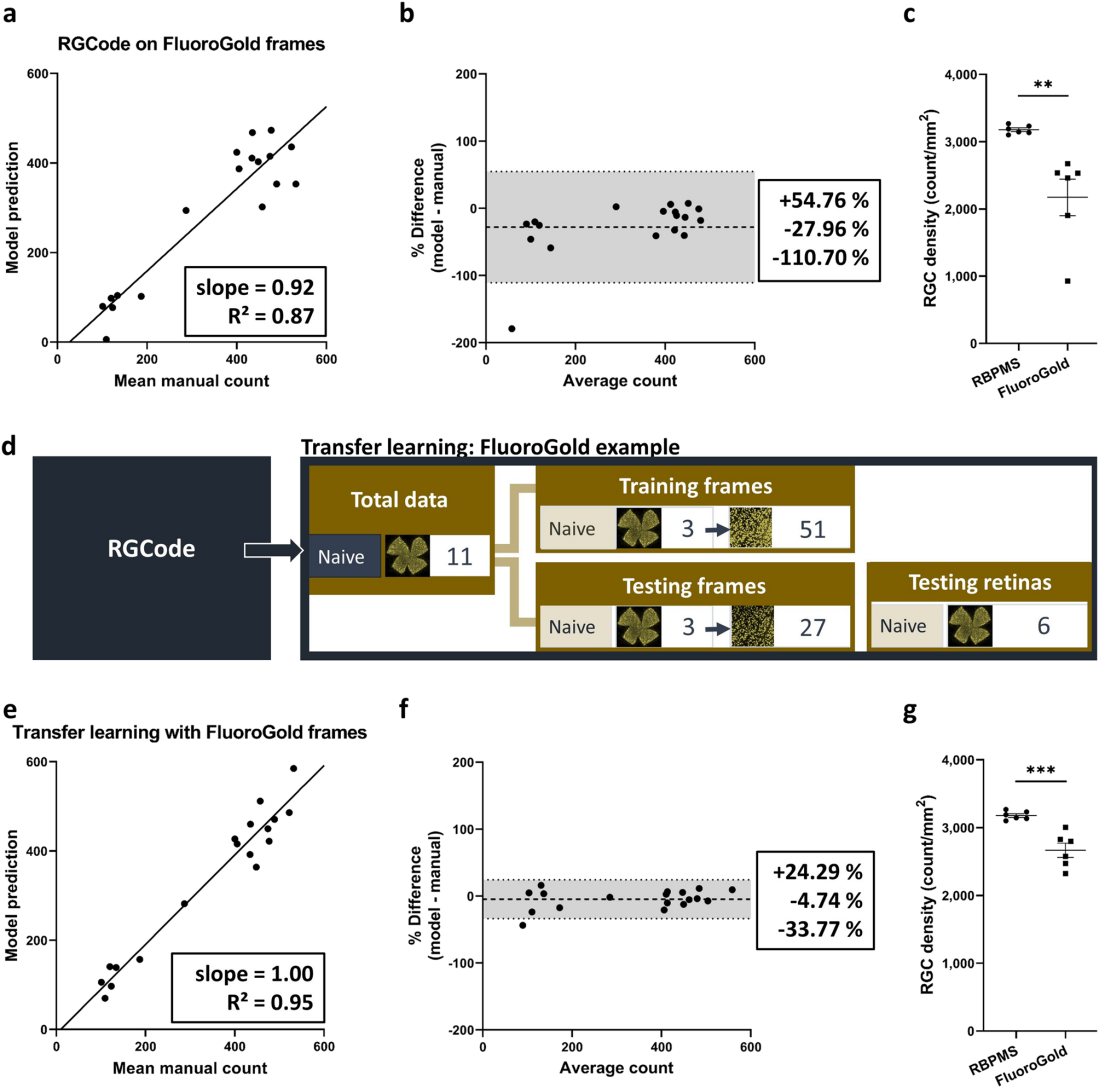
Transfer learning of RGCode for Fluorogold labelling. (**a**, **b**) Linear regression and Bland Altman analysis (mean bias ± 95% limits of agreement) after running RGCode on Fluorogold-labelled RGCs. Counting performance was considerably lower as compared to the RBPMS dataset, whereas a higher bias was observed. (**c**) the lower performance of RGCode on FluoroGold-traced flatmounts resulted in a high variability in density (mean ± SEM, unpaired, two-tailed *t* test, **p = 0.0042; ***p = 0.0007). (**d**) Composition of the training and testing dataset used for transfer learning. (**e**,**f**) Transfer learning of RGCode with a minimal set of new training data reveals a high accuracy with linear regression and Bland–Altman analysis. Mean bias ± 95% limits of agreement are depicted. (**g**) Transfer learning results in a lower variability in the RGC density measurements. As expected, the density of FluoroGold+ RGCs is significantly lower as compared to RBPMS+ ones (unpaired, two-tailed *t* test, ***p = 0.007).

After showing the performance of RGCode for automated counting and segmentation of RBPMS+ retinas, we next sought to explore whether our fully automated pipeline could be adapted to a different RGC label. As opposed to RBPMS, different RGC labelling techniques—such as the commonly used FluoroGold tracer—can pose different or additional difficulties. While training a new model from scratch requires a significant amount of data and time investment, transfer learning—i.e. the adaptation of a pretrained model to a new task—can reduce both investments^23^. Indeed, if the starting model has already been trained to process RGC labelling pictures, only the parameters of output layers of the neural network need to be fine-tuned to adapt it to a new dataset. As an example of transfer learning, we retrained our RBPMS counting model to count FluoroGold-traced RGCs in naive retinas. While the same neuronal cell type is labelled, the latter task is more challenging as retrograde tracing results in a more heterogeneous labelling of RGCs, with both bright, uniformly labelled cells and more faintly, unevenly labelled ones in different regions of the retina. The difficulties posed by this label were reflected in a lower performance, observed when running RGCode on FluoroGold-traced frames (Fig. 6a–c). Therefore, to apply transfer learning, we created two new datasets in the same manner as shown for RGCode. The training dataset—51 frames sampled from 3 naive retinas—comprised a total of 9,132 cells. The testing dataset consisted of 18 frames sampled from 3 naive retinas or a total of 6,134 cells. Additionally, 6 retinas were reserved to evaluate the performance on complete flatmounts (Fig. 6d). Despite this low amount of training data—51 against the 318 frames used to train RGCode—the linear regression and Bland–Altman analyses of the retrained model on FluoroGold-traced frames reached a satisfactory slope of best linear fit of 1.00, an $\hbox {R}{^2}$ of 0.95 and a bias of − 4.74%, with $\hbox {CI}_{95\%}$ [− 33.77,+ 24.19%] (Fig. 6e,f). Once again, Bland–Altman analysis of inter-operator bias showed comparable or higher biases and $\hbox {CI}_{95\%}$ (Supplementary Fig. S4). When run on entire flatmounts, the FluoroGold+ RGC counting model showed an RGC density of 2,668 ± 104 cells/$\hbox {mm}{^2}$. The resulting FluoroGold density statistically differs from the previously obtained RBPMS+ density, showing a FluoroGold labelling of 83.93% (Fig. 6g). Such result is in line with the expectations, given the applied unilateral retrograde tracing. Only the RGCs that project to the superior colliculus (± 90%)^24^—with exclusion of those projecting ipsilaterally (3–5%)^25^—are known to be labelled.

## Discussion

Although deep learning is not new, sufficient computational resources have only recently become available to begin to exploit this exciting technology. Current applications have primarily been focused around highly image-driven medical specialities^26^. As the eye is a very accessible organ for medical imaging, ophthalmology lends itself perfectly to the implementation of artificial intelligence approached for image segmentation^27^. Unsurprisingly, publications on “artificial intelligence in ophthalmology” have been booming since 2017, with more than 464 research articles published since this time (PubMed search conducted on May 29, 2020). In glaucoma, machine and deep learning algorithms have been developed for disease screening, diagnosis and monitoring of patients. As reviewed elsewhere^26,28^, most reports provide algorithms for the assessment of structural and/or functional changes after visual field tests, fundus imaging and optical coherence tomography. More recent advances include the analysis of contact lens sensor recordings^29^, retinal cell apoptosis via DARC technology^30^ and anterior chamber angle measurements using ultrasound biomicroscopy^31^ and optical coherence tomography^32^. To our knowledge, only two papers have been published in preclinical rodent research describing artificial intelligence algorithms. One—by Hedberg-Buenz et al.—describes the counting and classifying of retinal cells on H&E-stained uninjured and ONC-injured flatmounts using a random forest classifier algorithm^33^. The second, more recent one is a U-Net-based CNN approach published by Ritch et al. to calculate axon densities on rat semi-thin sections of uninjured and OHT-injured optic nerves^21^. To our knowledge, this model—called AxoNet—is the first counting model based on deep learning in ophthalmological animal research. Over the past 2 years, some deep learning counting algorithms have been published in neurobiology research for counting neurons^34–37^, microglia^22,35,38^ and astrocytes^39^ on stained sections of the rodent brain and spinal cord. While tackling a similar task, these methods differ from ours in the deep neural network architecture employed. Moreover, they do not combine cell counting with automated tissue segmentation to compute cellular densities.

In this paper we propose an all-in-one deep learning-based method to quantify RBPMS-labelled RGC densities. We use a combination of two U-Net models to automatically count cells and segment the retina. Our method, RGCode, achieves a high and consistent performance on both tasks, in every region of the retina, and for a variety of control and pathological conditions. The computed density of RBPMS-stained RGCs in uninjured retinas by RGCode was 3,179 ± 26 RGCs/$\hbox {mm}{^2}$, which is in line with other reports showing an average of $\approx 3,000$ RGCs/$\hbox {mm}{^2}$ after manual counts of RBPMS+ cells on flatmount retinas of C57Bl/6J mice^40,41^. For the glaucomatous conditions, outcomes of both injury paradigms strongly depend on the experimental design. Nevertheless, the observed 6.89 ± 1.90% reduction in RGC density in the microbead-induced OHT paradigm is in agreement with previous reports showing 7–9% RGC loss in CD57Bl/6 mice 4–6 weeks after microbead injection by manual counting of DAPI+^42^, neurobiotin-traced^43^ or Brn3a-immunopositive (Brn3a+) cells^44^. The reported RGC survival rate at 7 days after ONC injury (40.80 ± 1.96%) is also in accordance with studies using RGC recordings^9^, RBPMS immunohistochemistry^9,16,41,45^ and FluoroGold tracing^46–51^ in mice, all showing 20–50% RGC survival 1 week after nerve crush. Theoretically, RBPMS counts should be higher than Brn3a counts, as RBPMS should be expressed in all RGCs, whereas Brn3a is no longer seen as a pan-RGC marker and—at least for injured retinas—its expression diminishes when RGCs are coping with stress (cfr. Introduction). Indeed, comparing the current RBPMS counts with historical data from our lab, reveals a slightly higher RBPMS+ RGC density (3,179 ± 26 RGCs/$\hbox {mm}{^2}$) compared to Brn3a+ RGC density (2,954 Brn3a+ cells/$\hbox {mm}{^2}$) in naive retinas^52^. Furthermore, previous ONC studies with Brn3a labelling reported a total number of 1,150 Brn3a+ cells/$\hbox {mm}{^2}$ and an RGC survival of 38%^52^, which is slightly lower than the current results of our RBPMS model. Next and also in line with previous reports, isodensity maps of healthy retinas generated via RGCode illustrate a central-to-peripheral gradient in RGC density and a horizontal RGC-dense region called the visual streak^53–57^. A clear advantage of the isodensity maps is the swift detection of sectorial cell death, often described in experimental^58–60^ and genetic glaucoma models^56,61,62^. However, the injury paradigms used in this manuscript (microbead-induced OHT and ONC) are known to induce diffuse RGC loss across the entire retina with an even cell loss in all retinal quadrants^44,52,63^, as can be observed on the resulting isodensity maps. Last, the segmentation model shows an average retinal area of 14.18 ± 0.18 $\hbox {mm}{^2}$, which is comparable to values in literature^55,64^.

The major advantage of RGCode is its ability to perform the automated analysis on complete retinas, thereby eliminating the requirement of any—time consuming and error prone—user-input. This results in a 50 times faster quantification on a low-end computer as compared to an experienced counter. Being fully automated, RGCode is designed to make advanced image segmentation technologies accessible to a much wider audience, requiring only the startup of the program and the selection of the input folder containing the images, whereafter the full analysis runs in batch and takes only a few minutes per retina, with no variable tuning required. This is in stark contrast to manual counting on preselected frames that are currently still used by the majority of papers (>90% in the period 2010–2020). Manual counting is time-consuming and can potentially introduce a source of bias in the analysis, both at the frame selection and counting steps. While RGCode performed well in comparison to human operators, there still are minor shortcomings. Primarily, the model seems to have some problems identifying bright, large RGCs ($\ge 25 \,\upmu \hbox {m}$) in the retina. Fortunately, these represent only a minor fraction ($\approx 4\%$) of the total RGC population^8^ and render the influence on total count/density estimation negligible. Nevertheless, this issue can potentially be solved by changing the U-Net architecture to work with bigger receptive fields and higher spatial sampling. This would come at a price of an exponential increase in computational cost, which is currently still one of the major downsides of deep learning. To be easily accessible by everyone, the model should not require specialised computer hardware. Therefore, we aimed for a middle-ground between performance and computational cost, so that the analysis can be performed on consumer-grade computers. For the same reason, a complete retina is not analysed as a whole by RGCode, but is divided into tiles, of which the analysis results are next merged back together. This approach may generate edge-effect artefacts, an issue that was overcome by using a sliding-window approach with overlapping tiles, in which every tile shares part of its area with the adjacent ones. This prevents that cropping-artefacts—e.g. a cell falling on the border between two tiles—affect the output of the prediction model. The output of adjacent tiles is then merged in the stitching phase, hence significantly reducing edge-effects and rendering a final prediction output on the entire flatmount.

Finally, we have demonstrated the broad applicability of RGCode as it can be applied to differently imaged/scaled retinas and easily extended to other RGC labelling techniques. Indeed, we presented a transfer learning example of RGCode for FluoroGold-traced flatmounts, showing that retraining with a minimal amount of training data results in an excellent counting algorithm for this alternative application. Analysis of naive FluoroGold-traced retinas with this retrained model yielded an average density of 2,668 cells/$\hbox {mm}{^2}$—corresponding to a labelling of $\pm 84\%$ of the RBPMS+ RGCs—, which is comparable to previous literature reports showing 2,800–3,000 cells/$\hbox {mm}{^2}$^55,64,65^. Of note, the rather low values found here are related to the fact that we opted for a unilateral tracing from the superior colliculus, whereas others apply FluoroGold onto the transected optic nerve or to both superior colliculi. While RGCode was originally conceived for RGC density measurements and retrained for FluoroGold tracing, we believe that our pipeline can be retrained to count other retinal cell types and potentially even cells in other tissues. It should be noted that retraining might also be needed for RBPMS+ RGC counting of more severe phenotypes or when the input images are taken with substantially different scaling and/or magnification. Therefore, we provide training scripts along with the analysis software, so that other labs can fine-tune our models or train an entirely new one, after creating a new dataset tailored to their needs. Nonetheless, we predict that our pretrained models for RBPMS and FluoroGold can be used as-is by other researchers with similar data sets. In summary, RGCode brings the counting of RBPMS-stained retinas to the current state-of-the-art for Brn3a counting models and demonstrates breakthrough accuracy in fully automated murine RGC quantification with unprecedented ease and speed.

## Methods

### Experimental animals

Within this study, 8–12 weeks old C57Bl/6N (Charles River Laboratories, France) mice of either sex were used, which were housed under standard laboratory conditions. All experiments were approved by the Institutional Ethical Committee of KU Leuven and were in accordance with the European Communities Council Directive of 22 September 2010 (2010/63/EU).

### Surgical procedures

#### ONC

Unilateral ONC was performed as previously described^52^. Briefly, the optic nerve was exposed via an incision in the conjunctiva and crushed 1 mm from the globe with a Dumont #7 cross-action forceps (Fine Science Tools, Germany) for 5 s. Animals were anesthetised via an intraperitoneal injection of ketamine (75 mg/kg body weight, Nimatek, Eurovet) and medetomidine (1 mg/kg, Domitor, Pfizer), which was reversed after the surgical procedure by atipamezole (1 mg/kg, Antisedan, Pfizer). In addition, local analgesia (oxybuprocaïne 0.4%, Unicaïne, Théa) was applied on the eye before the surgery, and antibiotic ointment (tobramycin 0.3%, Tobrex, Alcon) was applied afterwards. Animals were euthanized 7 days post crush.

#### Microbead-induced OHT

Following the method described by Ito and Belforte et al.^66^, magnetic microbeads were injected in the anterior chamber of the right eye and re-positioned to the iridocorneal angle via a magnet. Anaesthesia was achieved using isoflurane (Iso-Vet 1000 mg/g, Eurovet; 4% for induction 1.5% for maintenance). Similar to the ONC procedure, local analgesia and antibiotic ointment were given. Tissues were harvested 5 weeks after injection.

#### Retrograde tracing from superior colliculus

The protocol for unilateral retrograde tracing from the superior colliculus was adapted from Nadal-Nicolás^67^. Using general anaesthesia (cfr. above), a cranial window of $2\times 2$ mm was made above the superior colliculus. After removing the dura mater, the exposed visual cortex was aspirated to expose the underlying superior colliculus. A sponge soaked in 4% hydroxystilbamidine (in saline with 10% dimethylsulfoxide, Life Technologies) was applied on the surface of the superior colliculus. The craniotomy was filled with elastomer (Kwik-cast, World Precision Instruments) and the skin was sutured. Animals were given meloxicam subcutaneously (5 mg/kg, Metacam, Boehringer-Ingelheim) for post-operative pain relief and were euthanized 6 days post-surgery.

### Tissue sampling and processing

All animals were sacrificed with an overdose of sodium pentobarbital (60 mg/kg, Dolethal, Vetoquinol), followed by transcardial perfusion with 0.9% saline and 4% paraformaldehyde. After enucleation, the eyes were post-fixed in 4% paraformaldehyde for 1 h and washed 3 times with PBS. This post-fixation step was repeated on the retinas after flatmounting. For the RBPMS immunohistochemical stainings, retinas from each treatment group (naive, OHT- and ONC-injured) were stained separately and in two different batches to account for technical variability. The retinal flatmounts were permeabilised by washing steps in 0.5% Triton X-100 in phosphate buffered saline (PBS) and a 15 min freeze-thaw step at $-80^{\circ }\hbox {C}$. Hereafter, retinas were incubated overnight with primary rabbit anti-RBPMS antibody (1/250, PhosphoSolutions) in PBS with 2% Triton X-100 and 2% pre-immune donkey serum. After multiple rinsing steps, a 2-h incubation with the secondary antibody (Alexa-488-conjugated donkey anti-rabbit IgG, 1/500, Life Technologies) was performed. Retinas were mounted with the anti-fading mounting medium Mowiol (10%, Sigma-Aldrich). Mosaic images of the entire retinal flatmounts were imaged with a wide-field epifluorescence microscope (Leica DM6) with a HC PL FLUOTAR L 20x/0.40 CORR objective, resulting in a resolution of 2.17 pixels/$\upmu \hbox {m}$. Confocal flatmount pictures were acquired on an Olympus FV1000 microscope, with a UPLSAPO 20X/0.75 objective, resulting in a resolution of 0.97 pixels/$\upmu \hbox {m}$.

### Creation of training and testing datasets

To train and test the RGC counting model, randomly selected 768 $\times$ 768 pixel frames (354 $\times$ 354 $\upmu$m) were sampled from predefined areas per retinal quadrant: optic nerve head (ONH), central, mid-peripheral, peripheral and border regions (see Supplementary Fig. S1). As such, data represent ± 10–15% of the total retina. The design and composition of the different datasets is explained above (cfr. Fig. 1). Counters were given three counting rules (Supplementary Fig. S2): (i) cells on the frames’ boundaries were only counted when more than half of the cell was visible; (ii) bright small cells, mostly visible in less dense regions, were excluded based on cell size and morphology (i.e. excluded when smaller than 9 $\upmu$m); (iii) very weakly stained cells were not counted. To increase the ease of manual counting, images were identically preprocessed in ImageJ^68^ by subtracting the background (“Subtract Background”, “rolling = 50”) and enhancing the local contrast (“Enhance Local Contrast (CLAHE)”, “blocksize = 127 histogram = 256 maximum = 3 mask = *None* fast_(less_accurate)”). Of note, for training and testing of the model, the original, unprocessed frames were provided as input, unless otherwise stated. To test robustness, testing frames were additionally subjected to random brightness shift, using the random_brightness function of the Python library tf.keras, with a range of (0.5, 1.5). Manual counting of RGCs was done with the Multi-point counting tool in ImageJ and manual annotations were transformed into training masks (Supplementary Fig. S8). To train the segmentation model, ground truth segmentation masks were created in ImageJ using the Polygon selection tool.

### Data augmentation and preprocessing

Before training, to minimise computational cost, the frames were cropped into 9 sub-frames (256 $\times$ 256 pixels) and re-scaled to half the size. Data augmentation techniques, as in random $\times$ and y coordinate shifts, flips and rotations, were used on the training frames to minimise overfitting and improve generalisation. For segmentation, whole-retina images were resized to 2048 $\times$ 2048 pixel pictures, which were then cropped in 16 sub-frames of 512 $\times$ 512 pixels. The same random transformations as for the counting frames were applied, with the addition of random shear angle transformations, with a maximum of 20 degrees.

### U-Net implementation(s) and training

The general architecture of both the counting and segmentation models is identical to the one described in the original U-Net paper by Ronneberger et al.^19^, with minor adaptations. Briefly, the architecture consists of 5 contraction and up-sampling blocks. Each contraction block consists of two convolutional sub-blocks (2D $3\times 3$ convolution, batch normalization and ReLU activation layers) followed by a $2\times 2$ Max Pooling layer and a Dropout layer with rate of 0.5, to minimize overfitting. The initial number of convolution filters (32) is doubled at each block. The up-sampling blocks consist of a 2D $3\times 3$ transposed convolutional layer, followed by a concatenation layer, a Dropout layer (0.5 rate) and the same convolutional sub-block as described above. The final output layer consists of a $1\times 1$ convolution with sigmoid activation. The loss function used for training is a combination of binary cross-entropy loss and Dice coefficient. We used the standard Keras Adam optimizer, with a learning rate of 10^–4^. The counting model was trained for 256 steps per epoch, for a total of 183 epochs i.e. when the validation loss stopped improving (Supplementary Fig. S9). We used a batch size of 32 and 64 validation steps. During training, the training dataset was randomly split between actual training and validation data, the latter being used to check the performance while training. We used a split of 50% to equally represent edge-cases in both datasets. Image augmentation, as described above, was applied on-the-fly during training. For segmentation, the training data was split 75/25% between training and validation. Similarly, the segmentation model was trained for 35 epochs (Supplementary Fig. S9), with a batch size of 16 and 64 validation steps. For transfer learning, the weights of a pre-trained model were loaded before starting the training, which was executed for 12 epochs, as described for the counting model.

### Post-processing and count prediction

Due to memory limitations, the model prediction could not be run on the complete retina. Therefore, we implemented a prediction on overlapping tiles to minimise edge-effects. Briefly, the complete retina was cropped in 256 $\times$ 256 pixel tiles with 12.5% overlap. The tiles were then re-scaled to 128 $\times$ 128 pixels and fed to the model. The predicted tiles were next re-stitched together and up-scaled to generate a prediction map of the same size of the input retina. This prediction was converted to a binary image. Subsequently, the retina was down-scaled once more to a 2048 $\times$ 2048 pixel image for segmentation, as high resolution is not required for this task. The total retina was divided in 25 tiles (512 $\times$ 512 pixels each) with 25% overlap. Identified RGCs outside of the retinal segmentation were discarded and the remaining ones were labelled and counted with the regionprops function of the scikit-image Python library (Version 0.16.1). The RGC density was calculated by dividing the RGC count by the segmented area. Isodensity maps are created by multivariate kernel density estimation with a Gaussian kernel with a bandwidth of $150 \,\upmu \hbox {m}$, using the kdeplot function of the seaborn library. The probability density function output is multiplied by the total RGC number to display true densities in cells/$\hbox {mm}{^2}$.

### Code and computer hardware

All the code used is available at GitLab (https://gitlab.com/NCDRlab/rgcode) and was written in Python 3.7. U-Net neural network architecture was implemented using the Tensorflow/Keras 2.0 libraries. Image and data pre- and post-processing was carried out using the Python libraries scikit-image, scikit-learn, OpenCV, numpy, scipy, matplotlib, seaborn, statsmodels and pandas. The models were trained on GPU clusters hosted by the Flemish Supercomputer Center (VSC) and validated on an office workstation computer (AMD Ryzen PRO 2700 CPU, 32GB RAM and Nvidia P1000 GPU). Nevertheless, the resulting counting program can be run on consumer-grade desktops and laptops.

### Statistical analysis

Plotting, linear regression and Bland–Altman analysis was done using the scipy Python library and GraphPad Prism (version 8.3, GraphPad Software). Intraclass correlation coefficient analysis was performed in the R software, using the psych library^69^. Normal distribution was tested via Shapiro–Wilk tests in GraphPad Prism, with a significance level of $\alpha = 0.05$. Other statistical tests were performed in Prism and are given in the figure legends. All data are described as mean ± SEM in the text.

## Supplementary information

Supplementary Information 1.

## Data Availability

All the relevant code is available on the public GitLab repository https://gitlab.com/NCDRlab/rgcode.
