# Supplementary material for: A novel retinal ganglion cell quantification tool based on deep learning
Source: Sci Rep. 2021 Jan 12;11:702. doi: 10.1038/s41598-020-80308-y (PMC7804414; doi:10.1038/s41598-020-80308-y)
Supplement: Supplementary file 1 — Supplementary Information 1. [file 41598_2020_80308_MOESM1_ESM.pdf]

A novel retinal ganglion cell quantification tool based on deep learning

Luca Masin[1,+], Marie Claes[1,+], Steven Bergmans[1], Lien Cools[1], Lien Andries[1], Benjamin M. Davis[2,3], Lieve Moons[1], and Lies De Groef[1,*]

[1]Neural Circuit Development and Regeneration Research Group, Department of Biology, KU Leuven, Leuven, Belgium
[2]Glaucoma and Retinal Neurodegenerative Disease Research Group, Institute of Ophthalmology, University College London, London, United Kingdom
[3]Central Laser Facility, Science and Technologies Facilities Council, UK Research and Innovation, Didcot, Oxfordshire, United Kingdom
[*]lies.degroef@kuleuven.be
[+]these authors contributed equally to this work

Supplementary information

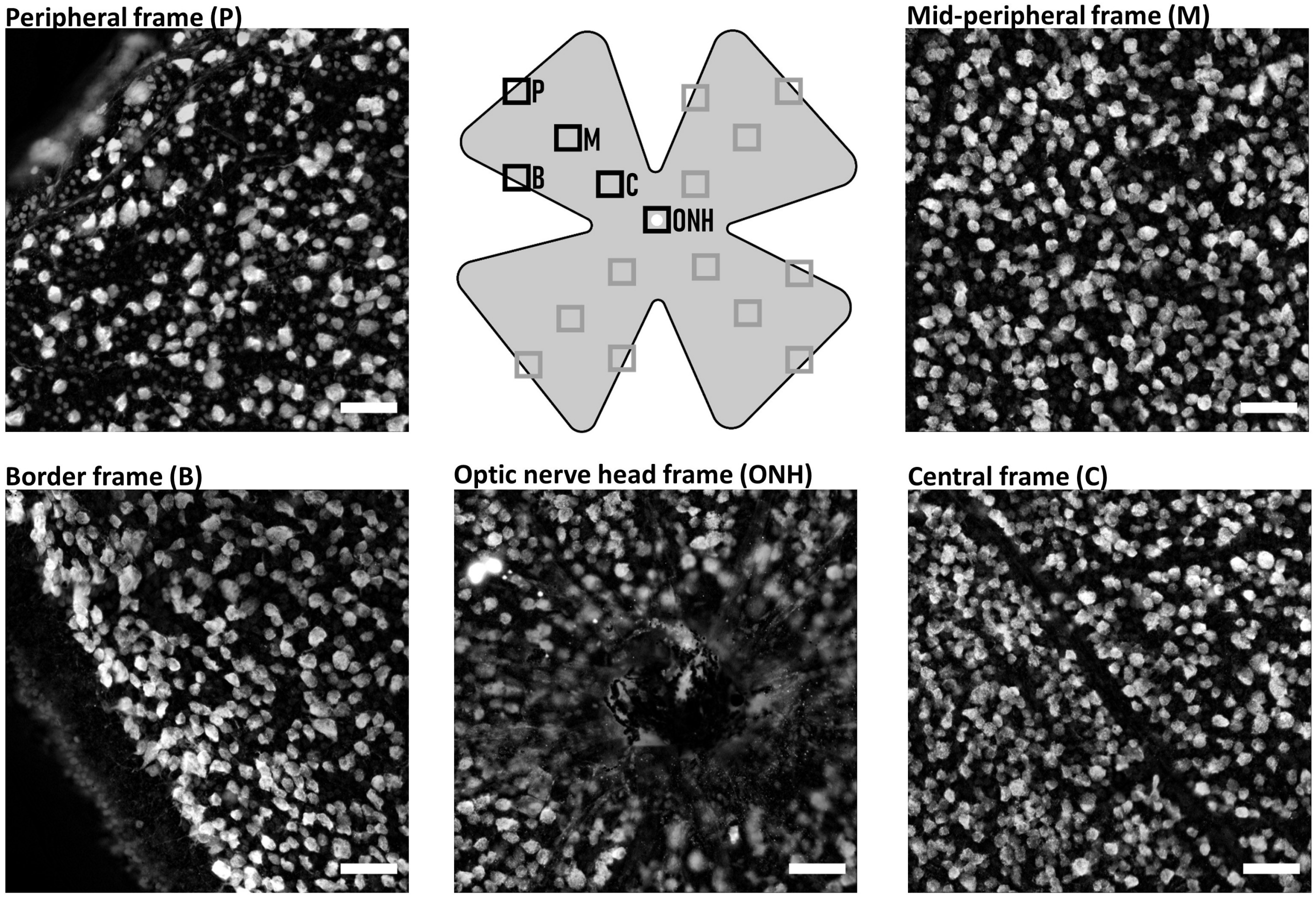

Supplementary Figure S 1. Representative photomicrographs of RBPMS immunostained counting frames (354 x 354 μm) in an uninjured retina. Locations include optic nerve head (ONH), central (C), mid-peripheral (M), peripheral (P) and border (B) frames. Scale bar = 50 μm.

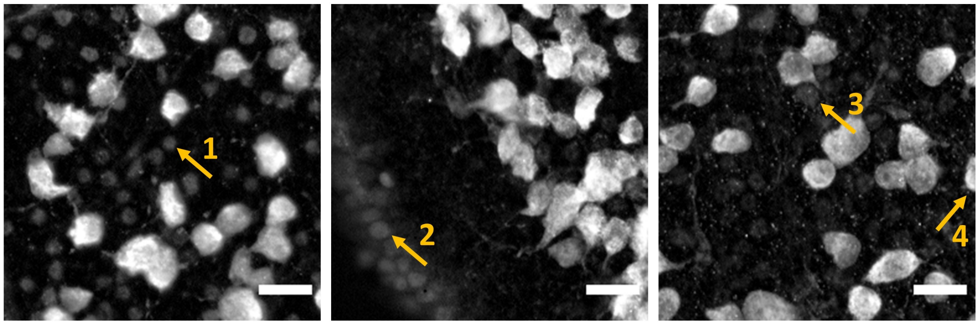

Supplementary Figure S 2. Magnified photomicrographs of the training frames, showing examples of the counting rules. Cells that were excluded are indicated by arrows. Arrow 1 denotes amacrine cells in the inner nuclear layer, only visible in the less dense peripheral retina. Border cells (arrow 2) and weakly stained cells (arrow 3) were not counted, as well as cells on the edge of the counting frame when clearly less than 50% of the cell was visible within the frame (arrow 4). Scale bar = 20 μm.

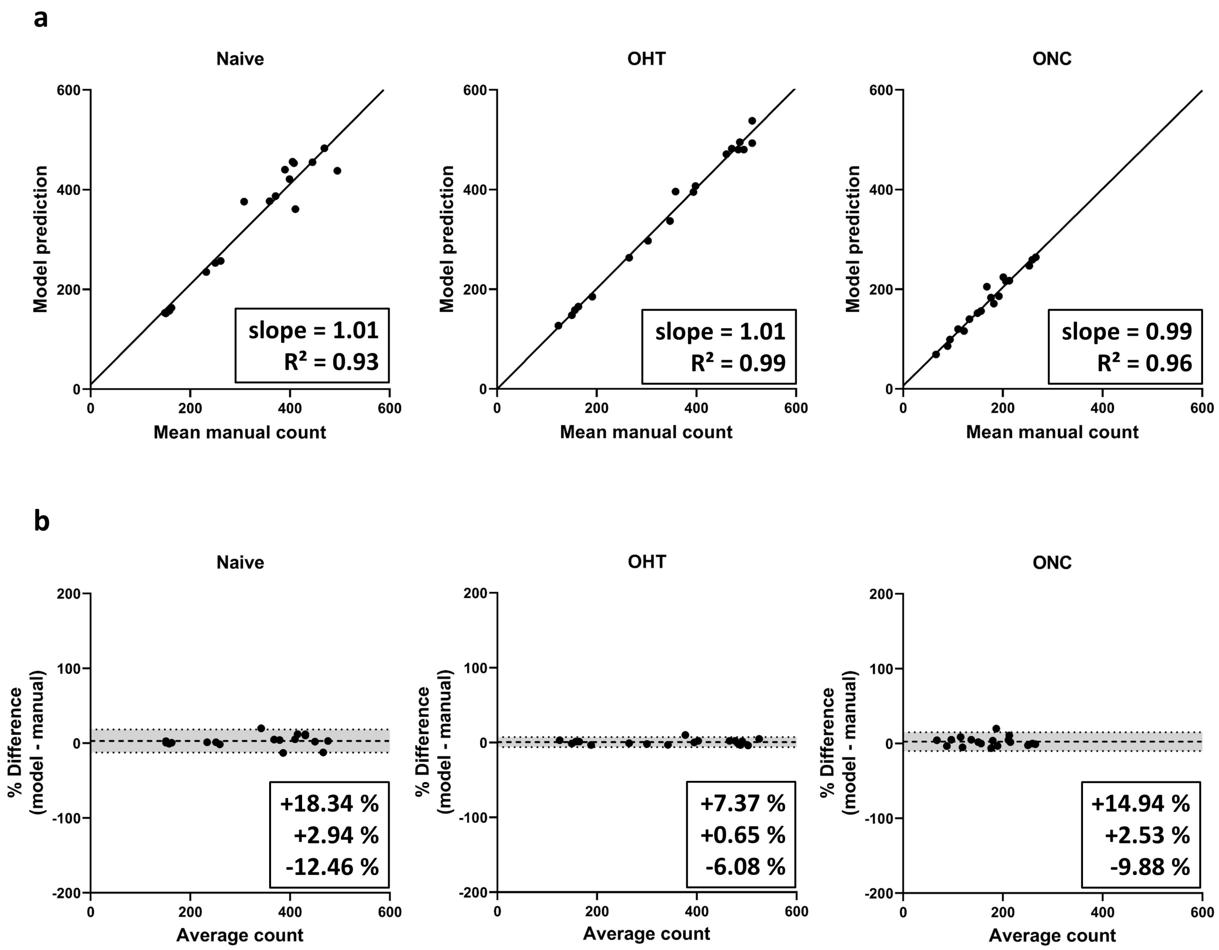

Supplementary Figure S 3. Performance of RGCode on retinal frames, shown per experimental condition. (a) Comparison of RGCode versus the average of manual counts via linear regression analysis. Best-fit linear regression correlation coefficient (R^2 and slope) are indicated. (b) Bland-Altman analysis of RGCode versus average manual counts, mean bias $\pm$ 95% limits of agreement are indicated. Key: Naive, uninjured retinas; OHT, 5 weeks post microbead-induced ocular hypertension retinas; ONC, 7 days post optic nerve crush injury retinas.

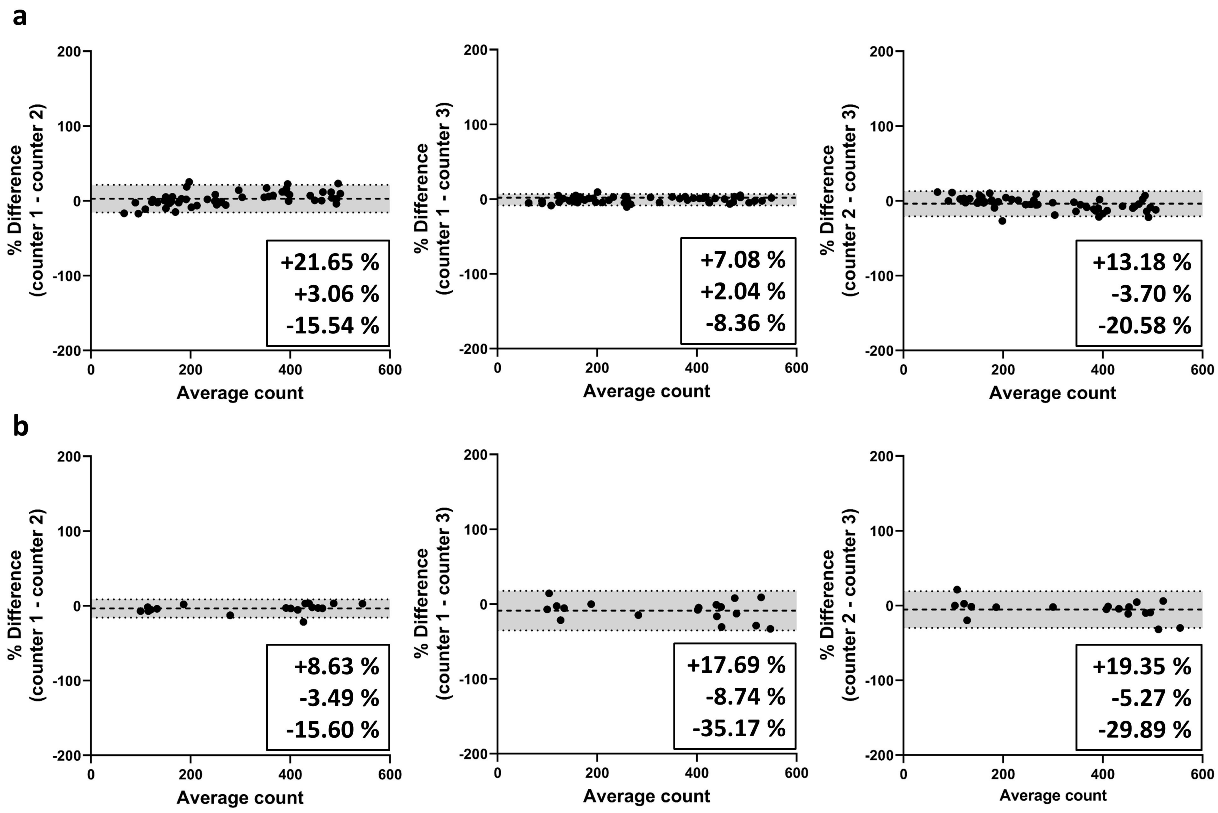

Supplementary Figure S 4. Bland-Altman plots comparing the inter-operator bias. (a) Comparison of manual annotations on sampling frames from RBPMS stained retinas. (b) Inter-operator bias of manual counting on FluoroGold-traced flatmounts. Mean bias ± 95% limits of agreement are indicated on the plots.

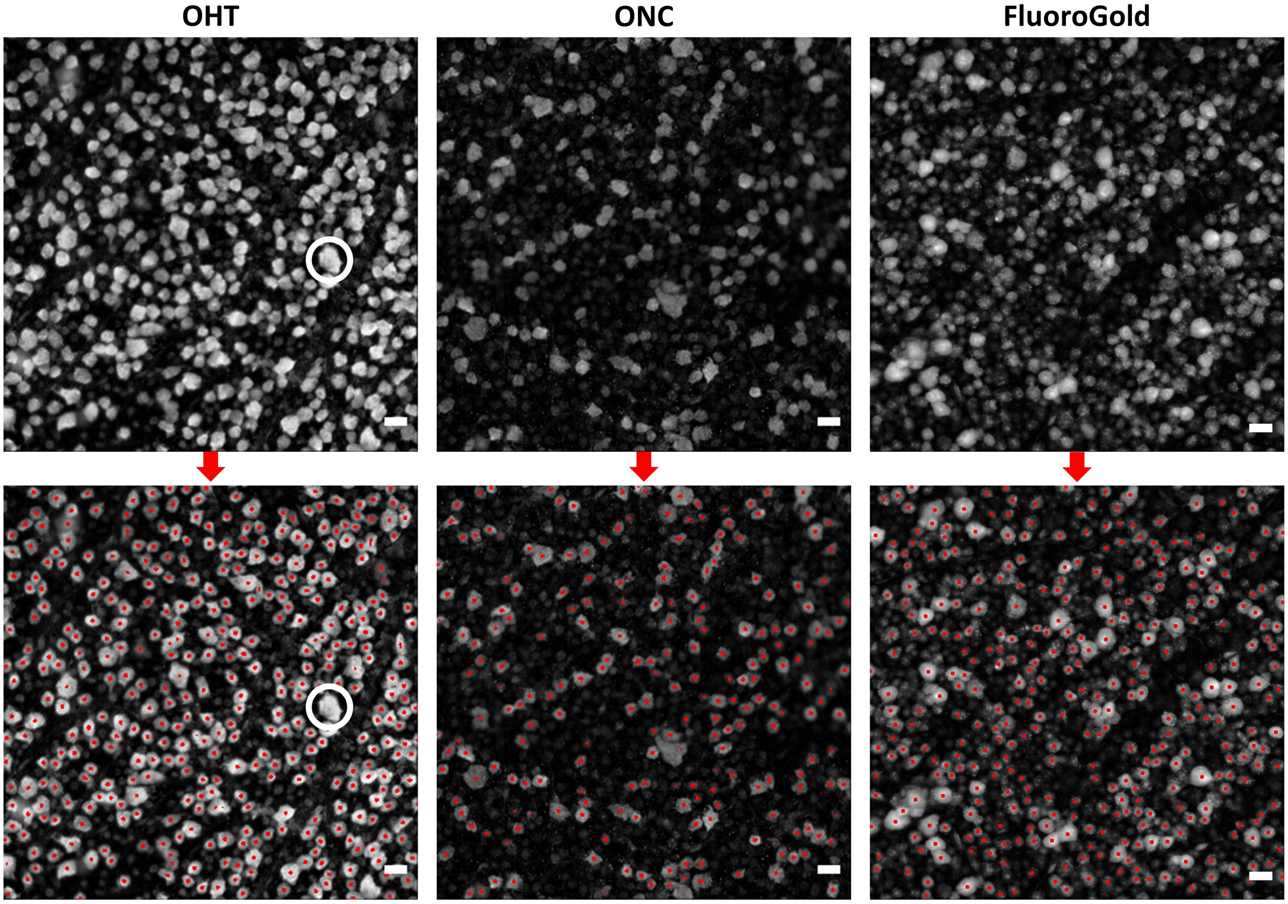

Supplementary Figure S 5. Representative examples showing the performance of the RGCode on different testing frames, sampled from the mid-peripheral retina. If any, limitations include the occasional misidentification of large ($\geq 25\mu$m), intensely stained RGCs, e.g. the encircled cell. Scale bar = 20 μm. Key: OHT, 5 weeks post microbead-induced ocular hypertension retinas; ONC, 7 days post optic nerve crush retinas; FluoroGold, 6 days post unilateral tracing from superior colliculus retinas.

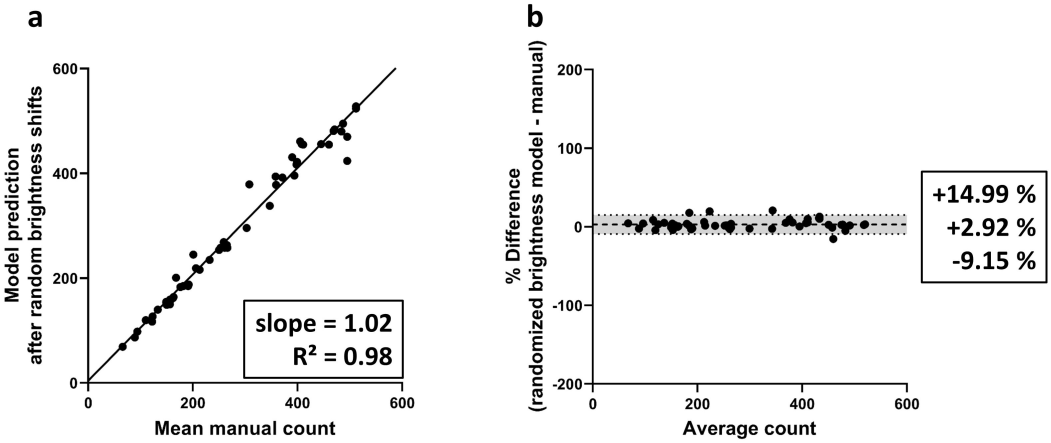

Supplementary Figure S 6. Linear regression and bias analysis of RGCode after randomized brightness shifts on testing dataset (a) Linear regression analysis for the average of the manual counts versus the randomized brightness model output. Best-fit linear regression correlation coefficient (R^2 and slope) are indicated. (b) Bland-Altman analysis of the automated counts after randomized brightness shifts compared to the operator average, showing mean bias $\pm$ 95% limits of agreement.

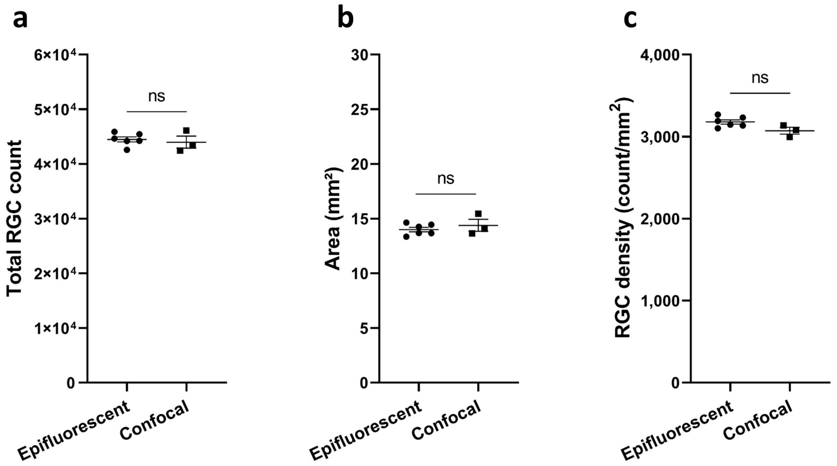

Supplementary Figure S 7. Comparison of the RGCode output on epifluorescence (n = 6) and confocal (n = 3) microscopy flatmount images (mean ± SEM). Total RGC count (a), retinal area (b) and RGC density (c) do not statistically differ upon changes in imaging technique (unpaired, two-tailed t-test).

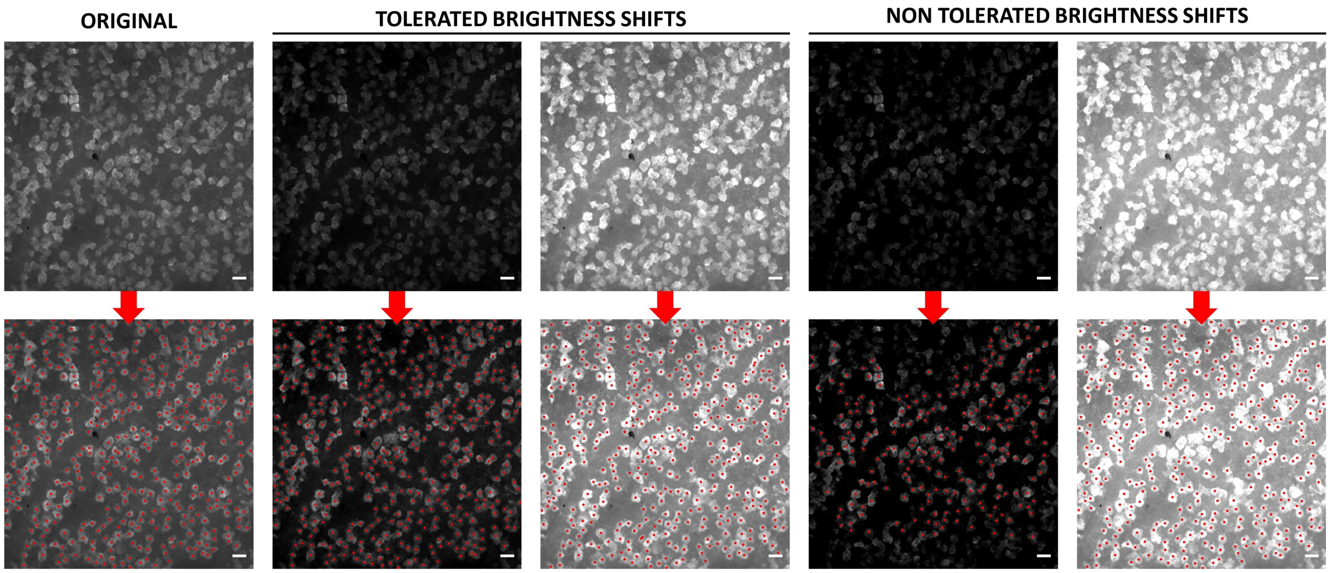

Supplementary Figure S 8. Examples showing different brightness shifts alongside the corresponding counts of RGCode, revealing the necessary quality of the input data in order to get reliable counts. Scale bar = 20 μm.

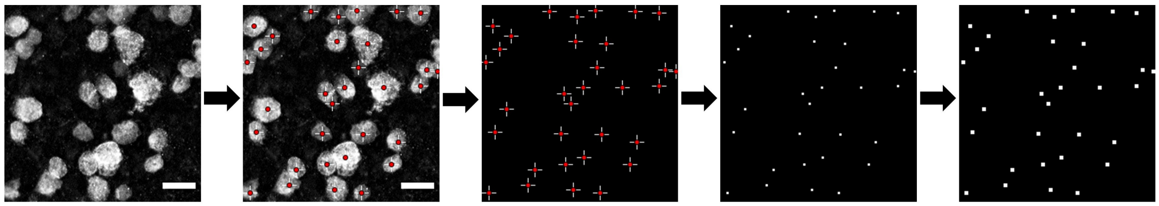

Supplementary Figure S 9. Generation of training masks in ImageJ. The manual counting points (red) defined by each counter are converted into a mask, which serves as input for the model. Scale bar = 20 μm.

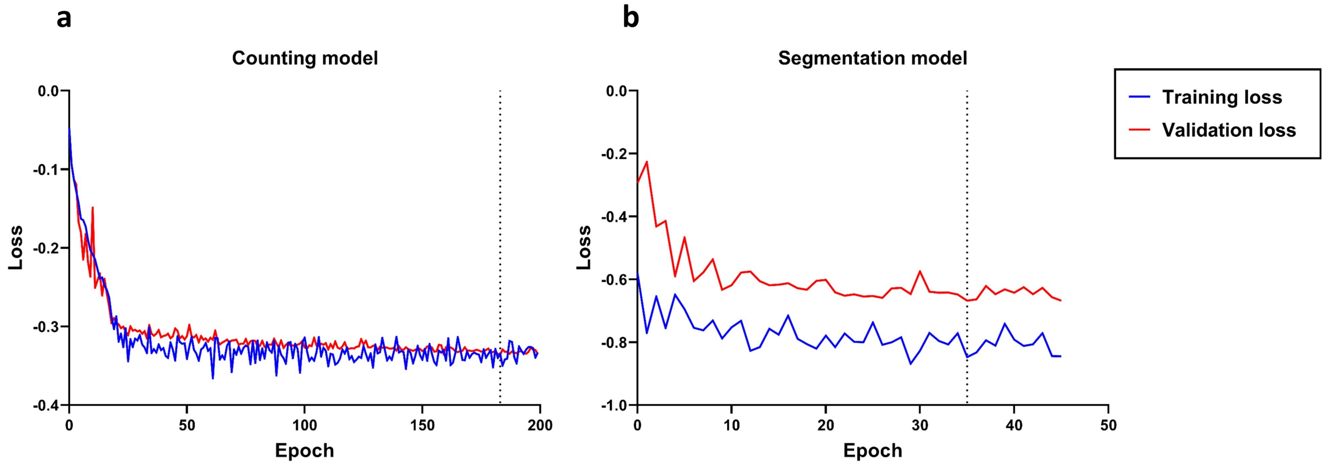

Supplementary Figure S 10. RGCode training curves. Training and validation loss for each training epoch for the counting (a) and segmentation (b) models. Validation loss does not diverge from the training one, indicating that the network is not overfitting. The model was saved at point of lowest loss (dotted lines), corresponding to epoch 183 for the counting model (a) and epoch 35 for the segmentation model (b).
